# Effects and safety of a CBD-rich *Cannabis sativa* oil in knee osteoarthritis: a double-blind, randomized, placebo-controlled trial – CANOA – cannabis for osteoarthritis

**DOI:** 10.3389/fphar.2025.1657065

**Published:** 2025-12-19

**Authors:** Andrés Mojoli, Osvaldo Haider, Yasmin Fakih, Maria Victoria Luz Gonçalves, Bruno Zepeda Rojas, Giovanna Xaia, Emanuelly Krefta, Maíra Assunção Bicca, Thiago Lopes de Mari, Charles Francisco Ferreira, Fernando Cezar-Dos-Santos, Aline Theodoro Toci, Francisney Pinto Nascimento

**Affiliations:** 1 Laboratório de Cannabis e Psicodélicos, Universidade Federal da Integração Latino-Americana (UNILA), Foz do Iguaçu, Brazil; 2 Laboratório Bioscientific, Curitiba, Brazil; 3 Department of Physiology, Universidade Federal do Rio Grande do Sul, Porto Alegre, Brazil; 4 Laboratório de Estudos Interdisciplinares do Meio Ambiente e Alimentos (LEIMAA), Universidade Federal da Integração Latino-Americana (UNILA), Foz do Iguaçu, Brazil

**Keywords:** osteoarthritis, pain, cannabinoids, cbd, WOMAC

## Abstract

Osteoarthritis is a common inflammatory and degenerative joint disease characterized by associated chronic pain, often ensuing to diminished quality of life. Current pain management options present small benefits and great side effects, driving interest in potential treatments such as cannabis, for its anti-inflammatory and analgesic effects. This trial aimed to assess the efficacy and safety of cannabidiol (CBD) in a full-spectrum cannabis oil in managing osteoarthritis-related pain.

**Methods:**

Osteoarthritis patients were randomized into either placebo or cannabis groups and monitored for 60 days. The cannabis group received a CBD daily oral dose of 45 mg. Primary outcome was determined by pain intensity measured utilizing the WOMAC, while secondary outcomes included the Visual Analogue Scale (VAS) Beck Depression Inventory (BDI), Pittsburgh Sleep Quality Index (PSQI) and the MC S12/PC S12 scores (mental and physical components of the quality of life SF-12 scale).

**Results:**

At the end of intervention (i.e. 60 days or trial end-point), both the placebo and cannabis groups exhibited comparable improvements in pain scores, with no statistically significant differences in pain intensity observed between groups. Likewise, secondary outcomes showed no significant differences between groups. Furthermore, the CBD-rich cannabis oil was well-tolerated, as no patients experienced any serious adverse events or clinically significant changes in serum biomarkers.

**Conclusion:**

CBD-rich cannabis oil treatment was well tolerated, with no serious adverse effects observed. However, this treatment did not demonstrate superiority over placebo in alleviating pain or improving secondary outcomes in osteoarthritis patients. Further multicentrical and larger trials are warranted to explore the efficacy of alternative dosages and/or formulations containing CBD, THC and other cannabinoids.

**Clinical Trial Registration:**

https://clinicaltrials.gov/study/NCT06588972 Identifier: [NCT06588972].

## 1 Introduction

Osteoarthritis is characterized by chronic pain and progressive inflammatory deformities of the joints, with the knee being the most frequently affected site (Prieto-Alhambra et al., 2014; Martel-Pelletier et al., 2016). This degenerative joint disorder is among the most prevalent causes of disability in the elderly, and due to the increasing prevalence of aging and obesity, it is currently estimated that approximately 250 million people worldwide are affected by the condition (Vos et al., 2016; Hunter and Bierma-Zeinstra, 2019).

Current therapies, including non-steroidal anti-inflammatory drugs (NSAIDs) and opioids, often provide limited pain relief and cause serious long-term side effects (Marcum and Hanlon, 2010). Of note, physiological and pathological inflammation and nociception processes are finely modulated by the endocannabinoid system.

The endocannabinoid system comprises endogenous lipid mediators and the enzymes responsible for their synthesis and degradation. The two main endocannabinoids are anandamide (AEA) and 2-arachidonoylglycerol (2-AG). AEA is synthesized by N-acyl phosphatidylethanolamine-specific phospholipase D (NAPE-PLD) and degraded by fatty acid amide hydrolase (FAAH), whereas 2-AG is produced by diacylglycerol lipases α and β (DAGLα and DAGLβ) and hydrolyzed mainly by monoacylglycerol lipase (MAGL). Both endocannabinoids exert their biological effects primarily through activation of the cannabinoid receptors CB1 and CB2 (Cristino et al., 2020). Notably, osteoarthritis individuals express in the synovial tissue both receptors (Richardson et al., 2008), that can be also exogenously activated by *Cannabis sativa* plant-derived molecules, collectively referred to as phytocannabinoids.

Among these phytocannabinoids, cannabidiol (CBD) has shown significant potential in alleviating chronic pain and inflammation across various pathological conditions (Manzanares et al., 2006; Vučkovic et al., 2018; Henson et al., 2022). However, clinical evidence regarding the efficacy of this isolated compound in osteoarthritis still remains limited and frequently inconsistent (Bebee et al., 2021; Dieterle et al., 2022; Schneider et al., 2022). In this context, several authors have proposed that the combination of phytocannabinoids naturally present in cannabis, such as delta-9-tetrahydrocannabinol (THC) and other non-major cannabinoids, may exert synergistic effects, thereby enhancing overall therapeutic potential. This phenomenon is well-described and known as the “entourage effect” (Russo, 2011; Anand et al., 2021), usually achieved when using full-spectrum cannabis oil.

On this basis, our trial was designed to evaluate the effects of a full spectrum CBD-rich cannabis oil in patients with osteoarthritis-associated knee pain. This randomized, double-blind and placebo-controlled clinical trial aimed to assess the treatment efficacy on pain and quality of life improvement. In addition, we report the safety of this type of treatment through the adverse events and biochemical parameters monitoring.

## 2 Methods

### 2.1 Cannabis based product

The commercial oil used in this trial consisted of a full-spectrum *Cannabis sativa* extract formulated with medium-chain triglycerides (MCT) as the excipient. The formulation was an amber-colored, oily liquid with a characteristic herbal odor and homogeneous appearance. The relative density of the product was 0.952 g/cm^3^, consistent with typical MCT-based preparations. Quantitative analysis of the main phytocannabinoids was performed using validated high-performance liquid chromatography (HPLC) (Dall’soto et al., 2025; F. et al., 2025) according to the guidelines of the National Institute of Metrology, Quality and Technology (Inmetro, 2020), and validation parameters included matrix effect, working range, linearity, limit of quantification, limit of detection, and homoscedasticity. Each parameter was evaluated to ensure the reliability and reproducibility of the quantitative results. Under these analytical conditions, CBD concentration was confirmed at 22.5 mg/mL, while Δ^9^-THC, terpenes, and other non-major cannabinoid concentrations in the oil remained below the analytical detection limit of 0.075 mg/mL, as measured by HPLC.

### 2.2 Clinical trial design

The CANOA trial was designed as a single-center, double-blind, randomized, and placebo-controlled trial conducted at the Laboratório de Cannabis e Psicodélicos, at the Universidade Federal da Integração Latino-Americana, in Brazil. Trial was approved by the Human Research Ethics Committee of the Universidade Estadual do Oeste de Paraná (CAAE:71278323.90000.0107) and followed the Good Clinical Practice (GCP) guidelines, the Declaration of Helsinki and the Brazilian resolution for clinical trials No. 466/12. The CANOA trial was registered on ClinicalTrials.gov (NCT06588972) and on Mendeley Data Repository (10.17632/fw6bftyrf2.1).

### 2.3 Participants

Patients diagnosed with knee osteoarthritis were enrolled from January 2024 until October 2024, after signing the informed consent form. Inclusion criteria were the following: knee osteoarthritis diagnosis established according to the American Rheumatism Association criteria (Altman et al., 1986), confirmed by clinical and radiographic findings and the presence of moderate to severe pain intensity (Visual Analogue Scale ≥5); age between thirty (30) and seventy (70) years-old; and for women of reproductive age, a negative Beta-HCG test and the use of a contraceptive method throughout the trial lasting for at least for 3 months after its conclusion. Exclusion criteria included patients with heart failure, hypertension, or any heart disease; substance use disorder; treatment with strong opioids; chronic kidney disease or liver failure; chronic inflammatory conditions; severe psychiatric disorders such as severe mood or psychotic disorders; current use of cannabinoids via any route of administration; pregnant or breastfeeding women.

### 2.4 Intervention

This was a double-blind, randomized and placebo-controlled clinical trial. Full-spectrum CBD-rich cannabis oil obtained from C*. sativa*–or MCT oil, as placebo–was administered orally twice per day (morning and evening) for 60 days (i.e., trial endpoint), a CBD daily dose of 45 mg. Patients were not required to be fasting at the oil administration time and continued their doctor-prescribed routine medications during the intervention period.

### 2.5 Randomization and blinding procedures

Randomization was stratified sequentially, first by pain intensity according to baseline WOMAC scores and subsequently by BMI categories, in order to ensure balanced allocation across clinically relevant strata. Within each stratum, participants were assigned to either the placebo or cannabis oil group using a computer-generated randomization list (random.org). Allocation concealment was maintained through the use of sealed, opaque, and sequentially numbered envelopes, handled by a research assistant not involved in participant recruitment or assessment. Both participants and researchers remained blinded to group allocation throughout the trial.

### 2.6 Outcome measures

#### 2.6.1 Primary outcome

The primary outcome was the change in pain levels, using the Likert version of the Western Ontario and McMaster Universities Osteoarthritis Index (WOMAC) and the pain domain of the WOMAC scale. Pain levels were categorized as 0-8 (mild), 9-14 (moderate), and 15-20 (severe), as described previously (Kapstad et al., 2008; Messier et al., 2022).

#### 2.6.2 Key secondary outcomes

Secondary outcomes included pain levels evaluated by Visual Analogue Scale (VAS), changes in quality of life assessed using the 12-Item Short-Form Health Survey (SF-12), focusing on the “maximum walking” and “activities of daily living” domains. Scores ranged from 0 (worst quality of life) to 100 (best quality of life). Depression levels were measured using the Beck Depression Inventory (BDI), with scores from 0-9 (no depression), 10-18 (mild to moderate depression), 19-29 (moderate to severe depression), and 30-63 (severe depression). Sleep quality was assessed using the Pittsburgh Sleep Quality Index (PSQI), with scores classifying sleep as good (0–4), poor (5–10), or indicating significant sleep disturbance (>10).

#### 2.6.3 Exploratory secondary outcomes

As a follow-up exploratory measure, we performed at trial commencement and endpoint a comprehensive metabolic and lipid panel analysis utilizing participants’ serums. For the metabolic panel the gamma-glutamyltransferase (GGT), aspartate aminotransferase (AST), alanine aminotransferase (ALT) and creatinine, whereas for the lipid panel the total cholesterol, high-density lipoprotein (HDL), low-density lipoprotein (LDL), very low-density lipoprotein (VLDL) and triglycerides were assessed. Values were expressed as U/L, units per liter.

### 2.7 Safety assessments

During this clinical trial, active monitoring of participants was conducted to detect any treatment-related adverse events. Participants were monthly interviewed to verify any unexpected effects and were encouraged to promptly report any adverse symptoms experienced during the clinical trial. Adverse events were documented and classified as non-serious or serious according to the International Council for Harmonisation (ICH) E2A guidelines (International Conference on Harmonisation of Technical Requirements for Registration of Pharmaceuticals for Human Use, 1994). The severity of adverse events was assessed using the *Udvalg for Kliniske Undersøgelser* (UKU) Side Effect Rating Scale, in which each adverse event is graded as mild, moderate, or severe according to its intensity and clinical impact (Lingjaerde et al., 1987).

### 2.8 Power and sample size considerations

The sample size was calculated using ANCOVA (fixed effects, main effects and interactions) in G*Power 3.1, which served as a conservative and appropriate approximation for our study design. The parameters used were: α = 0.05, β = 0.20 (power = 80%), effect size f = 0.42, and two groups. This effect size is comparable to the value reported in a meta-analysis of cannabinoid effects on pain (0.58) (Yanes et al., 2019), and was also consistent with the effect size (Wallace et al., 2015). The required sample size calculated under these conditions was 29 participants. Subsequently, it was possible to increase this number to 45 participants, enhancing the statistical power and reliability of the findings.

### 2.9 Statistical analysis

Continuous variables were expressed as means ± standard deviation (±SD) or as medians and 95% confidence intervals (95% CI, upper and lower limits), depending on data distribution as determined by the Shapiro-Wilk normality test. Categorical variables were presented as absolute (n) and relative frequencies (n%). Comparisons between continuous variables were performed using the independent samples Student’s t-test or the Mann-Whitney U test, as appropriate. Associations between categorical variables were evaluated using the Chi-square test with adjusted standardized residual analysis. All analyses were conducted according to a modified intention-to-treat (mITT) principle, which included all randomized participants who completed at least one post-baseline assessment. For longitudinal analysis of quantitative outcomes over time between treatment groups, we employed Generalized Estimating Equations (GEE) with a gamma distribution and log link function, considering repeated measurements across time points. The analytical model accounted for within-subject correlations and provided robust estimations of group-by-time effects. Missing data were handled using the mean imputation method. Fisher’s exact test was employed to compare the proportions of adverse events between groups, with multiple testing controlled via the Benjamini–Hochberg procedure. Risk ratios (RR) were calculated as the ratio of event incidence in the cannabis group to that in the placebo group, with 95% confidence intervals (CIs) estimated using the logarithmic method based on the standard error of log(RR). For events with zero cases in one or both groups, RR and CIs were not computed. All statistical analyses were performed using SPSS version 18.0, and statistical significance was set at *p* ≤ 0.05.

## 3 Results

### 3.1 Patients baseline characteristics

**FIGURE 1:**
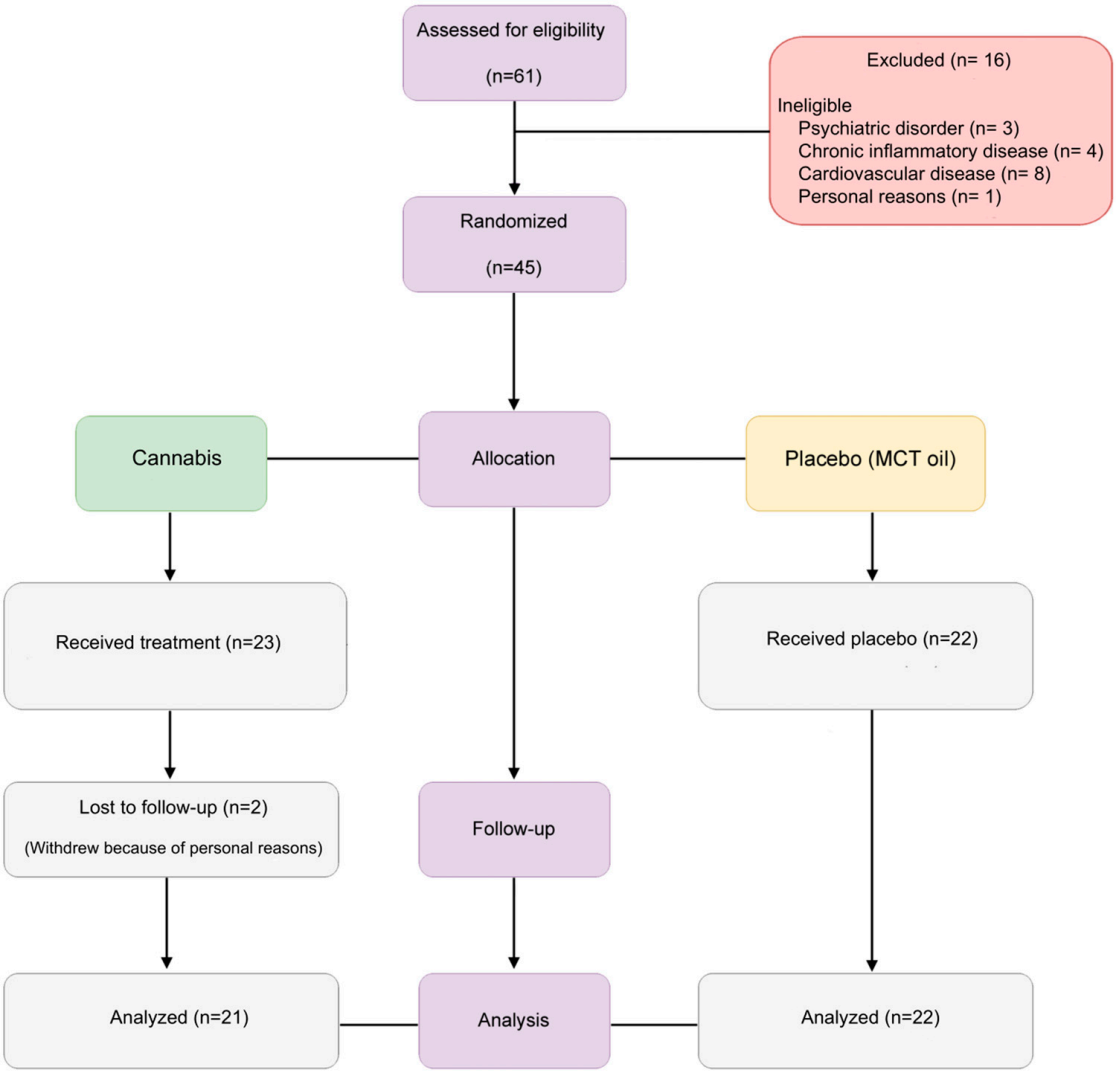
Participant flow through the CANOA trial.

Sixty-one patients averaged 61.91 years and diagnosed with osteoarthritis were screened. Most of these patients were women and white, representing 88.88% and 66.66% respectively, of the trial population. Following exclusion and inclusion criteria screening, forty-five patients were randomized to either placebo or cannabis group, comprehending patients of similarly distributed age, sex and BMI baseline characteristics (Figure 1). Both groups were well balanced in respect to concomitant medication being taken. In the placebo group, the most commonly used medications were NSAIDs (50.0%), paracetamol or dipyrone (40.91%), and weak opioids (22.73%). Similarly, in the cannabis group, NSAIDs (43.48%) and paracetamol or dipyrone (39.13%) were also the most frequent medications, while weak opioids accounted for 8.70% of concomitant treatments. Corticosteroid use was comparable between groups (13%). Regarding lifestyle factors, no participants were smokers, and only a single individual in the cannabis group reported alcohol consumption.

With respect to pain assessment at baseline, total WOMAC scores were 66.82 (95% CI: 59.30–74.33) and 69.48 (62.84–76.11) for the placebo and cannabis groups, respectively. Similarly, the WOMAC pain subscale scores were 13.45 (95% CI: 11.69–15.22) for the placebo group and 13.78 (95% CI: 12.51–15.06) for the cannabis group. Finally, the VAS scores were 8.50 (95% CI: 7.91–9.09) and 8.04 (95% CI: 7.22–8.86) for the placebo and cannabis groups, respectively.

In this regard, primary, secondary, and exploratory outcomes were comparable between groups, indicating homogeneity of the study population before intervention. These and other baseline characteristics of patients included in our clinical trial are shown in Table 1. Unlike patients in the placebo group, two patients from the cannabis group withdrew because of personal reasons. A schematic flow chart summarizing randomization procedures is presented on Figure 1.

**TABLE 1:** Baseline parameters.

Characteristics	Placebo	Cannabis	*p* value
N	22	23	
Age, y mean [CI]	61.91 [56.65–67.17]	61.96 [57.27–66.64]	0.98
Sex, n (%)			
Women	21 (95.45)	19 (82.61)	0.18
Men	1 (4.55)	4 (17.39)	0.18
BMI, mean [CI]	31.84 [29.38–34.31]	31.61 [28.92–34.30]	0.89
Monthly income, n (%)			
up to 1 minimum wage	5 (22.73)	4 (17.39)	0.72
1–2 minimum wages	9 (40.91)	6 (26.09)	0.36
2–7 minimum wages	8 (36.36)	10 (43.48)	0.77
>7 minimum wages	-	3 (13.04)	0.11
Race, n (%)			
White	16 (72.73)	14 (60.87)	0.39
Multiracial (*pardo*)	4 (18.18)	8 (34.78)	0.21
Black	1 (4.55)	1 (4.35)	1
Prefer not to answer	1 (4.55)	-	0.49
Education level, n (%)			
Less than high school	11 (50.0)	10 (43.48)	0.77
High school graduate	7 (31.82)	5 (21.74)	0.52
College or professional degree	4 (18.18)	8 (34.78)	0.20
Currently therapy, n (%)			
Paracetamol and dipyrone	9 (40.91)	9 (39.13)	1.00
Antidepressants	4 (18.18)	7 (30.44)	0.49
NSAIDs	11 (50.0)	10 (43.48)	0.76
Corticosteroids	3 (13.64)	3 (13.04)	1.00
Weak opioids	5 (22.73)	2 (8.70)	0.21
Antiepileptics	3 (13.64)	9 (23.13)	0.50
Smoker, n	0	0	
Alcohol user, n (%)	0	1 (4.35)	0.48
WOMAC score, mean [CI]	66.82 [59.30–74.33]	69.48 [62.84–76.11]	0.58
WOMAC pain score, mean [CI]	13.45 [11.69–15.22]	13.78 [12.51–15.06]	0.75
VAS score, mean [CI]	8.50 [7.91–9.09]	8.04 [7.22–8.86]	0.35
BDI score, mean [CI]	13.32 [9.89–16.75]	15.30 [10.52–20.09]	0.49
PSQI score, mean [CI]	9.32 [7.45–11.19]	9.7 [8.22–11.17]	0.74
SF-12 score			
PC S12	27.82 [24.22–31.42]	27.46 [25.40–29.53]	0.85
MC S12	42.62 [37.15–48.08]	39.18 [32.85–45.50]	0.39
Serology			
ALT U/L, mean [CI]	22.33 [19.06–25.61]	22.41 [18.09–24.73]	0.68
AST U/L, mean [CI]	26.14 [23.07–29.20]	24.70 [21.83–27.56]	0.48
GGT U/L, mean [CI]	31.23 [10.31–52.14]	34.30 [245.71–43.90]	0.77
Creatinine mg/dL, mean [CI]	0.97 [0.88–1.05]	0.94 [0.87–1,01]	0.64
Cholesterol mg/dL, mean [CI]	192.8 [180.2–205.4]	204.3 [187.2–221.4]	0.26
HDL mg/dL, mean [CI]	58.23 [51.78–64.67]	55.59 [50.29–60.89]	0.51
LDL mg/dL, mean [CI]	100.1 [87.26–112.9]	113.0 [97.78–128.2]	0.18
VLDL mg/dL, mean [CI]	34.45 [26.98–41.93]	38.79 [28.90–48.68]	0.47
Triglycerides	172.1 [134.9–209.2]	193.8 [144.4–243.2]	0.65

N, number; y, years; CI, confidence interval; BMI, body mass index; NSAID, Non-Steroidal Anti-Inflammatory Drug; WOMAC, Western Ontario and McMaster Universities Osteoarthritis Index; VAS, visual analogue scale; BDI, beck depression inventory; PSQI, pittsburgh sleep quality index; SF-12, 12-Item Short-Form Health Survey; PC S12, Physical Component Summary; MC S12, Mental Component Summary; ALT, alanine aminotransferase; AST, aspartate aminotransferase; GGT, Gamma-Glutamyl Transferase; HDL, High-Density Lipoprotein; LDL, Low-Density Lipoprotein; VLDL, Very Low-Density Lipoprotein; U/L, units per liter; mg/dL, *Milligrams per Deciliter*.

### 3.2 Primary outcome

**FIGURE 2:**
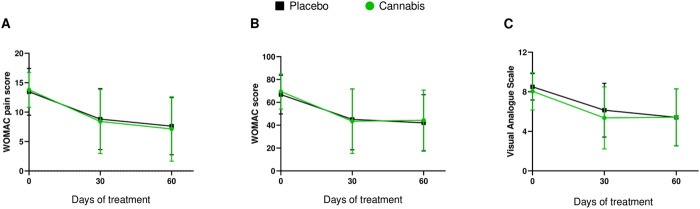
Progression of pain levels over time, measured using **(A)** the WOMAC Pain, **(B)** WOMAC and **(C)** the VAS scales in patients with knee osteoarthritis. Data represents mean ± standard deviations.

As shown in Figures 2A,B, no statistically significant differences were observed between the placebo and cannabis groups regarding mean pain intensity, as measured by both the WOMAC pain subscale and the total WOMAC scores, 60 days after treatment. At the trial end point, fifteen patients of each group reported some decrease in these two scales, representing 68.2% and 71.4% of the patients in the placebo and cannabis group, respectively. Although both groups improved over time (time effect), this improvement occurred to a similar extent in each group, with no significant group-by-time interaction. Table 2 summarizes treatment-induced findings for WOMAC pain subscale. At the trial end point, the mean reduction was 8.35 (95% CI: 6.59–10.58) for the placebo group and 7.60 (95% CI: 5.66–10.21) for the cannabis group. Additionally, total WOMAC scores at 60 days were 42.56 (95% CI: 33.79–53.59) and 42.72 (95% CI: 33.00–55.30) for the placebo and cannabis groups, with a mean group difference of −0.17 (95% CI: 22.27−21.94; p = 0.86) (Table 2). Effect size analysis (Cohen’s *d*) revealed negligible group differences across primary outcomes, consistent with the non-significant statistical results.

**TABLE 2:** Primary and secondary outcomes.

Outcomes	Placebo		Cannabis		Mean difference	Cohen’s d values	*p* value
	T0	T60	T0	T60	(95% CI)		
WOMAC pain score	13.45	8.35	13.78	7.60	0.74 (−3.74–5.22)	0.17	0.794
WOMAC score	66.82	42.56	69.48	42.72	−0.17 (−22.27–21.94)	0.09	0.864
VAS score	8.50	5.57	8.04	5.37	0.2 (−2.17–2.58)	0.01	0.663
BDI score	13.32	6.77	15.30	7.22	−0.45 (−7.09–6.20)	0.03	0.872
PSQI score	9.32	6.14	9.70	6.34	−0.20 (−3.73–3.33)	0.08	0.752
SF-12 score							
PC S12	27.81	36.91	27.46	37.86	−0.96 (−12.50–10.58)	0.05	0.872
MC S12	42.62	56.00	39.18	54.28	1.72 (−8.31–11.75)	0.16	0.680

WOMAC, Western Ontario and McMaster Universities Osteoarthritis Index; VAS, visual analogue scale; BDI, beck depression inventory; PSQI, pittsburgh sleep quality index; SF-12, 12-Item Short-Form Health Survey; PC S12, Physical Component Summary; MC S12 Mental Component Summary.

### 3.3 Key secondary outcomes

As observed for primary outcomes, at the trial endpoint, no treatment-induced effects were reached at any secondary outcomes with negligible effect sizes (Cohen’s d), as also shown in Table 2. Mean reduction in VAS score was 5.57 (95% CI: 4.57–6.79) for the placebo group and 5.37 (95% CI: 4.34–6.63) for the cannabis group, being 0.2 (95% CI: 2.17−2.58; p = 0.66) the mean group difference (Figure 2C). BDI mean scores did not show significant variation between groups, with values of 6.77 (95% CI: 3.93–11.67) and 7.22 (95% CI: 5.13–10.17) for the placebo and cannabis groups (mean group difference −0.45 [95% CI: 7.09−6.20]; p = 0.87). Comparable results were achieved among PC S12 and MC S12 values of the SF-12, being 36.91 (95% CI: 31.58–43.13) and 56.00 (95% CI: 51.6–60.7) the scores for the placebo group, and 37.86 (95% CI: 33.07–43.35) and 54.28 (95% CI: 49.5–59.4) registered for the cannabis group, respectively.

### 3.4 Exploratory secondary outcomes

During the clinical trial, nine different serum parameters were monitored: GGT, AST, ALT, creatinine, total cholesterol, HDL, LDL, VLDL. At the trial endpoint, we did not observe any changes in any of the serum biomarkers considered throughout the trial. A complete description of these parameters is presented on Table 3.

**TABLE 3:** Comprehensive metabolic and lipid panel serum values.

Biomarker	Placebo		Cannabis	
	T0	T60	T0	T60
GGT	31.23 (10.31–52.14)	37.1 (6.716–67.47)	34.3 (24.71–43.90.)	34.57 (23.41–45.73)
AST	26.14 (23.07–29.20)	23.33 (21.20–25.47)	24.7 (21.83–27.56)	24.35 (21.74–26.96)
ALT	22.33 (19.06–25.61)	19.8 (16.25–23.35)	21.41 (18.09–24.73)	21.1 (17.43–24.77)
Creatinine	0.97 (0.88–1.05)	1.05 (0.9449–1.154)	0.94 (0.87–1.01)	0.98 (0.91–1.05)
Cholesterol	192.8 (180.2–205.4)	192.8 (178.8–206.8)	204.3 (187.2–221.4)	193.2 (177.2–209.3)
HDL	58.23 (51.78–64.67)	49.1 (44.36–53.83)	55.59 (50.29–60.89)	48.43 (43.75–53.11)
LDL	100.1 (87.26–112.9)	108.3 (94.04–122.6)	113 (97.78–128.2)	109 (96.37–121.6)
VLDL	34.45 (26.98–41.93)	35.46 (26.30–44.62)	38.79 (28.90–48.68)	36.6 (26.42–46.78)
Triglycerides	172.1 (134.9–209.2)	177.3 (131.5–223.1)	193.8 (144.4–243.2)	183 (132.1–233.9)

GGT, gamma-glutamyltransferase; AST, aspartate aminotransferase; ALT, alanine aminotransferase; HDL, high-density lipoprotein; LDL, low-density lipoprotein; VLDL, very low-density lipoprotein; U/L, units/liter. Data are shown as mean and 95% confidence interval.

### 3.5 Adverse events

No serious adverse effects were reported throughout the entire clinical trial (Table 4), as well as no treatment-induced significant adverse events were found. All recorded adverse events were mild in severity. At the end of the treatment, ten types of adverse events in the placebo group and eleven types in the cannabis group were reported. More precisely, adverse events were recorded for 22 patients from the placebo group and 20 patients from the cannabis group. Among all adverse events, constipation (21.7%), weight gain (30.4%), weight loss (21.7%) and decreased salivation (34.7%) were the most common adverse events reported in the cannabis group. In fact, a similar number of patients reported weight gain as much as weight loss, for both groups. Decreased salivation was the event most described affecting six patients (27.2%) in the placebo group and eight patients (34.7%) in the cannabis group. Nonetheless, no statistical differences between groups were observed.

**TABLE 4:** Adverse events registered during the clinical trial.

Event	Placebo no (%)	Cannabis no (%)	Risk ratio (RR)	95% CI	*p* value
Difficulty concentrating	0 (0%)	0 (0%)	–	–	1.00
Drowsiness	3 (13.6%)	3 (13.0%)	0.96	0.22–4.18	1.00
Memory	4 (18.1%)	3 (13.0%)	0.72	0.18–2.84	1.00
Tremor	1 (4.5%)	0 (0%)	0.21	0.01–4.15	1.00
Nausea/Vomiting	0 (0%)	1 (4.3%)	–	–	0.69
Diarrhea	1 (4.5%)	1 (4.3%)	0.96	0.06–15.00	1.00
Increased salivation	0 (0%)	1 (4.3%)	–	–	0.69
Decreased salivation	6 (27.2%)	8 (34.7%)	1.28	0.53–3.12	0.77
Constipation	2 (9.0%)	5 (21.7%)	2.41	0.52–11.17	0.49
Dizziness	6 (27.2%)	2 (8.6%)	0.32	0.07–1.43	0.52
Palpitation/Tachycardia	1 (4.5%)	2 (8.6%)	1.90	0.18–20.04	0.89
Erythema	0 (0%)	0 (0%)	–	–	1.00
Itching	0 (0%)	0 (0%)	–	–	1.00
Weight gain	4 (18.1%)	7 (30.4%)	1.68	0.57–4.94	0.59
Weight loss	3 (13.6%)	5 (21.7%)	1.59	0.42–6.03	0.69

## 4 Discussion

This clinical trial assessed the use of a full spectrum CBD-rich cannabis oil to treat osteoarthritis-associated knee pain. After 60 days of treatment (trial endpoint), improvements were observed in primary and secondary outcomes for both the placebo and cannabis groups, with no significant differences between them. Of note, cannabis extract administration did not induce any adverse events, as confirmed by both clinical and laboratory assessments. Considering current literature, we are originally reporting, under these clinical trial conditions, the use of a full-spectrum *C. sativa* extract for the treatment of osteoarthritis-associated knee pain.

Pain, as defined by the international association, is an unpleasant sensory and emotional experience, associated with, or resembling that associated with, actual or potential tissue damage. More specifically, osteoarthritis-associated pain intensity (which comprises sensory components) and perception (which comprises emotional bias) are very complex phenomena, posing challenges in measurements that include participants’ optimistic attitudes, recall bias, or even their desire to please the researcher. Furthermore, the Hawthorne effect can influence pain assessment, potentially complicating results interpretation, which might explain why improvements are observed even in the placebo group, potentially masking the difference between the treated and no treated groups (Wise et al., 2010; Berthelot et al., 2011).

In our trial, no significant cannabis-induced improvement was observed in pain levels when compared to the placebo group, as assessed using the WOMAC scale (the primary outcome). Corroborating, another clinical trial with osteoarthritis patients has failed to show CBD-induced pain alleviation. Patients with osteoarthritis-induced knee pain treated with 600 mg/day of isolated CBD for 8 weeks presented no significant amelioration when compared with placebo (Pramhas et al., 2023). Here, we employed a lower dose of 45 mg of CBD per day to minimize potential toxic effects, while also considering the possible entourage effect commonly observed with full-spectrum (containing multiple cannabinoids) cannabis extracts (Russo, 2011; Anand et al., 2021). Additionally, cannabinoids usually exhibit biphasic effects, displaying a non-linear dose-response relationship where lower doses may exert therapeutic benefits, while higher concentrations can lead to reduced efficacy (Shustorovich et al., 2024). In even lower dosages, 10–20 mg/day of CBD, patients with hand or psoriatic arthritis did not experience pain reduction (Vela et al., 2022). Of note, augmented CBD concentrations have been linked to elevated hepatic aminotransferase levels in treated patients (Devinsky et al., 2018; Pramhas et al., 2023). Overall, CBD only does not seem to be efficient to control pain levels, as assessed by others and our primary outcome measures.

Likewise, the secondary outcomes here addressed showed no significant treatment-induced improvement over time, as reported by no significant differences between the placebo and cannabis groups. Comorbidities such as depression and anxiety not only exacerbate pain levels but also increase disability in arthritis, ensuing diminished quality of life in affected patients (Creamer et al., 1999; Lin et al., 2003; Loggia et al., 2008; Duica et al., 2020). For both groups, depression symptoms and sleep difficulties -assessed using the BDI and the PSQI, respectively -, showed notable amelioration. In addition, both general health status and health-related quality of life also improved, with no significant differences observed between the groups. These outcomes could also be explained by the same factors possibly influencing the primary outcome, including but not limited to the Hawthorne effect, expectancy bias and the sense of support developed with the clinical trial investigator. Established patient-researcher relationships present the potential to create emotional states involving a sense of being cared for and supported, which can significantly impact treatment outcomes (Kelley et al., 2014), while emotional distress is known to conjointly amplify the painful experience. In this sense, this overall emotional distress mitigation induced by the trial might also have played a role in the primary outcome observed changes. We acknowledge that further trials with larger sample sizes are needed to generalize our findings, as well as the addressing of different cannabinoids combinations, concentrations and ratios.

Different cannabinoids combinations and ratios could lead to a fuller analgesic effect. For instance, the analgesic properties of less expressed cannabinoids as cannabigerol and cannabichromene have already been described (Morales et al., 2017; Wen et al., 2023; Sepulveda et al., 2024). We here employed a full-spectrum oil with virtually no THC, leaving for debate the potential analgesic properties of THC for osteoarthritis therapy, as well as the urge to investigate the effects of oils containing both CBD and THC. This clinical trial proves wrong the existent non-clinical literature showing CBD possesses anti-inflammatory and pain-modulating properties in osteoarthritis (Gamble et al., 2018; O’Brien and McDougall, 2018; Verrico et al., 2020; Mejia et al., 2021; Vaughn et al., 2021), and still, the possible mechanisms involved in this phenomenon have eluded study.

Most CBD mechanisms of action are non-cannabinoid mediated at low doses. CBD acts as a modest agonist of serotoninergic 5-HT1A receptors, modulating pain perception (Russo et al., 2005) and also binds to and desensitizes the transient receptor potential vanilloid (TRPV1), disrupting pain signaling. Additionally, CBD affects endocannabinoid biodisponibility by inhibiting the reuptake of AEA and its hydrolysis by FAAH (Bisogno et al., 2001). Meanwhile, the combination of CBD and THC as analgesic therapy remains controversial in literature. While some studies suggest that CBD may synergistically enhance THC’s analgesic effects (Russo, 2011), others report that the combination is ineffective, with analgesic effects observed only with isolated THC (Gorbenko et al., 2024). THC produces analgesic effects by modulating supraspinal pathways involved in the perception and processing of pain at brain level (Manzanares et al., 2006). Unlike CBD, it regulates pain and inflammation by directly activating CB1 and CB2 receptors (Henson et al., 2022), reducing pain perception and unpleasantness (Lee et al., 2013). In this trial, THC and other cannabinoid levels in the oil were below the detection limit of our methodology. That said, it is unlikely that THC exerted any significant effect in these patients, given its practically minimal dosage. Even though we assume the observed outcomes and adverse events in this clinical trial are primarily attributable to CBD we can not completely rule out THC participation.

THC and CBD adverse events follow a dose-dependent manner and differ in pattern. The former is mainly associated with dizziness, drowsiness, dry mouth, vomiting, and cognitive impairments (Gottschling et al., 2020). Increased appetite is also attributed to THC -induced CB1 receptor activation, which in turn induces augmented food intake and may promote weight gain (Koch and Matthews, 2001; Wiley et al., 2005). In contrast, CBD is more frequently linked to diarrhea, somnolence, reduced appetite, vomiting, and elevations in liver enzymes (Gottschling et al., 2020). Our 2 months treatment did not lead to any alterations in biochemical parameters, nor did it result in any psychotropic or serious adverse effects. The most frequently reported adverse events in the cannabis group included decreased salivation, weight variations (gain or loss), and constipation. At the trial endpoint, weight gain (30.4%) and weight loss (21.7%) were similarly reported among cannabis-treated patients, with no statistically significant differences compared to the placebo group. Collectively, our findings are consistent with findings reported in other studies (Consroe et al., 1991; Naftali et al., 2017; Irving et al., 2018) and highlight the extremely safe profile of cannabis treatment in patients with knee osteoarthritis.

## 5 Limitations

This trial presents limitations that should be recognized. First, THC and other minor cannabinoids were below the detection limit of our analytical method. Therefore, the lack of efficacy of treatment may be related to the low cannabinoid dosage—particularly undetectable THC content—in the administered oil. In this context, it becomes critical to provide a detailed phytochemical characterization of cannabis oils, addressing the concentration and potential contribution of other cannabinoids present in full-spectrum formulations. Moreover, only one specific CBD-rich formulation and dosage were evaluated; future studies should explore different cannabinoid combinations, concentrations, and ratios to better determine their therapeutic potential. Finally, the relatively small sample size limits the generalizability of our findings, underscoring the need for larger, multicentric clinical trials to confirm and expand upon these results.

## 6 Conclusion

In this clinical trial, the use of a CBD-rich cannabis oil for osteoarthritis-induced knee pain did not result in significant improvement compared to the placebo treatment. Nevertheless, the treatment demonstrated a favorable safety and tolerability profile, with no major adverse or psychotropic effects observed over the 2-month intervention period. These findings support the safety and tolerability of medicinal cannabis in our conditions.

We highlight the need for future studies using different combinations of CBD and THC–possibly with other cannabinoids–and most importantly the use oils with higher THC content, as well as longer follow-up periods and larger and multicentrical populations to better determine potential cannabinoid efficacy for pain management associated with osteoarthritis.

## Data Availability

All data from this trial are publicly available in the Mendeley Repository, doi: 10.17632/fw6bftyrf2.2. Available at: https://data.mendeley.com/datasets/fw6bftyrf2/2.

## References

[B1] Altman R. Asch E. Bloch D. Bole G. Borenstein D. Brandt K. (1986). Development of criteria for the classification and reporting of osteoarthritis. Classification of osteoarthritis of the knee. Diagnostic and therapeutic criteria committee of the American rheumatism association. Arthritis Rheum. 29, 1039–1049. 10.1002/art.1780290816 3741515

[B2] Anand U. Pacchetti B. Anand P. Sodergren M. H. (2021). Cannabis-based medicines and pain: a review of potential synergistic and entourage effects. Pain Manag. 11, 395–403. 10.2217/pmt-2020-0110 33703917

[B3] Bebee B. Taylor D. M. Bourke E. Pollack K. Foster L. Ching M. (2021). The CANBACK trial: a randomised, controlled clinical trial of oral cannabidiol for people presenting to the emergency department with acute low back pain. Med. J. Aust. 214, 370–375. 10.5694/mja2.51014 33846971

[B4] Berthelot J. M. Le Goff B. Maugars Y. (2011). The hawthorne effect: stronger than the placebo effect? Jt. Bone Spine 78, 335–336. 10.1016/j.jbspin.2011.06.001 21742532

[B5] Bisogno T. Hanuš L. De Petrocellis L. Tchilibon S. Ponde D. E. Brandi I. (2001). Molecular targets for cannabidiol and its synthetic analogues: effect on vanilloid VR1 receptors and on the cellular uptake and enzymatic hydrolysis of anandamide. Br. J. Pharmacol. 134, 845–852. 10.1038/sj.bjp.0704327 11606325 PMC1573017

[B6] Consroe P. Laguna J. Allender J. Snider S. Stern L. Sandyk R. (1991). Controlled clinical trial of cannabidiol in Huntington’s disease. Pharmacol. Biochem. Behav. 40, 701–708. 10.1016/0091-3057(91)90386-g 1839644

[B7] Creamer P. Lethbridge-Cejku M. Costa P. Tobin J. D. Herbst J. H. Hochberg M. C. (1999). The relationship of anxiety and depression with self-reported knee pain in the community: data from the Baltimore longitudinal study of aging. Arthritis Care Res. 12, 3–7. 10.1002/1529-0131(199902)12:1<3::aid-art2>3.0.co;2-k 10513484

[B8] Cristino L. Bisogno T. Di Marzo V. (2020). Cannabinoids and the expanded endocannabinoid system in neurological disorders. Nat. Rev. Neurol. 16, 9–29. 10.1038/s41582-019-0284-z 31831863

[B9] Dall’soto L. E. Pauli K. B. Fakih Y. R. C. Castañeda J. B. B. Toci A. T. de Carvalho M. A. F. (2025). A high-dose THC cannabis oil improves motor and non-motor symptoms in parkinson’s disease: a case report. Rev. Bras. Farmacogn. 35, 1046–1053. 10.1007/s43450-025-00675-3

[B10] Devinsky O. Patel A. D. Thiele E. A. Wong M. H. Appleton R. Harden C. L. (2018). Randomized, dose-ranging safety trial of cannabidiol in Dravet syndrome. Neurology 90, e1204–e1211. 10.1212/WNL.0000000000005254 29540584 PMC5890607

[B11] Dieterle M. Zurbriggen L. Mauermann E. Mercer-Chalmers-Bender K. Frei P. Ruppen W. (2022). Pain response to cannabidiol in opioid-induced hyperalgesia, acute nociceptive pain, and allodynia using a model mimicking acute pain in healthy adults in a randomized trial (CANAB II). Pain 163, 1919–1928. 10.1097/j.pain.0000000000002591 35239547 PMC9982727

[B12] Duica L. Szakács J. Călina S. S. (2020). Study on the correlation between knee osteoarthritis and anxiety in patients aged over 55. Balneo Res. J., 95–104. 10.12680/balneo.2020.323

[B13] F. A. M. L. G. B. R G.-J. S. K. I. M. (2025). Effectiveness of full spectrum cannabis extracts in the treatment of chronic pain: an open label study. J. Pain Palliat. Care Pharmacother. 39, 346–352. 10.1080/15360288.2025.2517778 40526158

[B14] Gamble L. J. Boesch J. M. Frye C. W. Schwark W. S. Mann S. Wolfe L. (2018). Pharmacokinetics, safety, and clinical efficacy of cannabidiol treatment in osteoarthritic dogs. Front. Vet. Sci. 5, 1–9. 10.3389/fvets.2018.00165 30083539 PMC6065210

[B15] Gorbenko A. A. Heuberger J. A. A. C. Klumpers L. E. de Kam M. L. Strugala P. K. de Visser S. J. (2024). Cannabidiol increases psychotropic effects and plasma concentrations of Δ9-Tetrahydrocannabinol without improving its analgesic properties. Clin. Pharmacol. Ther. 116, 1289–1303. 10.1002/cpt.3381 39054656

[B16] Gottschling S. Ayonrinde O. Bhaskar A. Blockman M. D’agnone O. Schecter D. (2020). Safety considerations in cannabinoid-based medicine. Int. J. Gen. Med. 13, 1317–1333. 10.2147/IJGM.S275049 33299341 PMC7720894

[B17] Henson J. D. Vitetta L. Hall S. (2022). Tetrahydrocannabinol and cannabidiol medicines for chronic pain and mental health conditions. Inflammopharmacology 30, 1167–1178. 10.1007/s10787-022-01020-z 35796920 PMC9294022

[B18] Hunter D. J. Bierma-Zeinstra S. (2019). Osteoarthr. *Lancet* 393, 1745–1759. 10.1016/S0140-6736(19)30417-9 31034380

[B19] Inmetro (2020). Orientação sobre validação de métodos analíticos, DOQ-CGCRE-008. Available online at: https://www.gov.br/cdtn/pt-br/centrais-de-conteudo/documentos-cgcre-abnt-nbr-iso-iec-17025/doq-cgcre-008/view.

[B20] International Conference on Harmonisation of Technical Requirements for Registration of Pharmaceuticals for Human Use (1994). Harmonised tripartite guideline: clinical safety data management: definitions and standards for expedited reporting E2a. *Effic. Guidel.* , 1–12.

[B21] Irving P. M. Iqbal T. Nwokolo C. Subramanian S. Bloom S. Prasad N. (2018). A randomized, double-blind, placebo-controlled, parallel-group, pilot study of cannabidiol-rich botanical extract in the symptomatic treatment of ulcerative colitis. Inflamm. Bowel Dis. 24, 714–724. 10.1093/ibd/izy002 29538683

[B22] Kapstad H. Hanestad B. R. Langeland N. Rustøen T. Stavem K. (2008). Cutpoints for mild, moderate and severe pain in patients with osteoarthritis of the hip or knee ready for joint replacement surgery. BMC Musculoskelet. Disord. 9, 55. 10.1186/1471-2474-9-55 18426591 PMC2386464

[B23] Kelley J. M. Kraft-Todd G. Schapira L. Kossowsky J. Riess H. (2014). The influence of the patient-clinician relationship on healthcare outcomes: a systematic review and meta-analysis of randomized controlled trials. PLoS One 9, e94207. 10.1371/journal.pone.0094207 24718585 PMC3981763

[B24] Koch J. E. Matthews S. M. (2001). Delta9-tetrahydrocannabinol stimulates palatable food intake in lewis rats: effects of peripheral and central administration. Nutr. Neurosci. 4, 179–187. 10.1080/1028415x.2001.11747361 11842887

[B25] Lee M. C. Ploner M. Wiech K. Bingel U. Wanigasekera V. Brooks J. (2013). Amygdala activity contributes to the dissociative effect of cannabis on pain perception. Pain 154, 124–134. 10.1016/j.pain.2012.09.017 23273106 PMC3549497

[B26] Lin E. H. B. Katon W. Von Korff M. Tang L. Williams J. W. Kroenke K. (2003). Effect of improving depression care on pain and functional outcomes among older adults with arthritis: a randomized controlled trial. JAMA 290, 2428–2429. 10.1001/jama.290.18.2428 14612479

[B27] Lingjaerde O. Ahlfors U. G. Bech P. Dencker S. J. Elgen K. (1987). The UKU side effect rating scale. A new comprehensive rating scale for psychotropic drugs and a cross-sectional study of side effects in neuroleptic-treated patients. Acta Psychiatr. Scand. Suppl. 334, 1–100. 10.1111/j.1600-0447.1987.tb10566.x 2887090

[B28] Loggia M. L. Mogil J. S. Bushnell M. C. (2008). Experimentally induced mood changes preferentially affect pain unpleasantness. J. Pain 9, 784–791. 10.1016/j.jpain.2008.03.014 18538637

[B29] Manzanares J. Julian M. Carrascosa A. (2006). Role of the cannabinoid system in pain control and therapeutic implications for the management of acute and chronic pain episodes. Curr. Neuropharmacol. 4, 239–257. 10.2174/157015906778019527 18615144 PMC2430692

[B30] Marcum Z. A. Hanlon J. T. (2010). Recognizing the risks of chronic nonsteroidal anti-inflammatory drug use in older adults. Ann. Longterm. Care 18, 24–27. 10.1017/S0031182010001277.Lipid 21857795 PMC3158445

[B31] Martel-Pelletier J. Barr A. J. Cicuttini F. M. Conaghan P. G. Cooper C. Goldring M. B. (2016). Osteoarthritis. Nat. Rev. Dis. Prim. 2, 16072. 10.1038/nrdp.2016.72 27734845

[B32] Mejia S. Duerr F. M. Griffenhagen G. McGrath S. (2021). Evaluation of the effect of cannabidiol on naturally occurring osteoarthritis-associated pain: a pilot study in dogs. J. Am. Anim. Hosp. Assoc. 57, 81–90. 10.5326/JAAHA-MS-7119 33450016

[B33] Messier S. P. Beavers D. P. Queen K. Mihalko S. L. Miller G. D. Losina E. (2022). Effect of diet and exercise on knee pain in patients with osteoarthritis and overweight or obesity: a randomized clinical trial. JAMA 328, 2242–2251. 10.1001/jama.2022.21893 36511925 PMC9856237

[B34] Morales P. Hurst D. P. Reggio P. H. (2017). Molecular targets of the phytocannabinoids: a complex picture. Prog. Chem. Org. Nat. Prod. 103, 103–131. 10.1007/978-3-319-45541-9_4 28120232 PMC5345356

[B35] Naftali T. Mechulam R. Marii A. Gabay G. Stein A. Bronshtain M. (2017). Low-dose cannabidiol is safe but not effective in the treatment for crohn’s disease, a randomized controlled trial. Dig. Dis. Sci. 62, 1615–1620. 10.1007/s10620-017-4540-z 28349233

[B36] O’Brien M. McDougall J. J. (2018). Cannabis and joints: scientific evidence for the alleviation of osteoarthritis pain by cannabinoids. Curr. Opin. Pharmacol. 40, 104–109. 10.1016/j.coph.2018.03.012 29635215

[B37] Pramhas S. Thalhammer T. Terner S. Pickelsberger D. Gleiss A. Sator S. (2023). Oral cannabidiol (CBD) as add-on to paracetamol for painful chronic osteoarthritis of the knee: a randomized, double-blind, placebo-controlled clinical trial. Lancet Reg. Heal. - Eur. 35, 100777. 10.1016/j.lanepe.2023.100777 38033459 PMC10682664

[B38] Prieto-Alhambra D. Judge A. Javaid M. K. Cooper C. Diez-Perez A. Arden N. K. (2014). Incidence and risk factors for clinically diagnosed knee, hip and hand osteoarthritis: influences of age, gender and osteoarthritis affecting other joints. Ann. Rheum. Dis. 73, 1659–1664. 10.1136/annrheumdis-2013-203355 23744977 PMC3875433

[B39] Richardson D. Pearson R. G. Kurian N. Latif M. L. Garle M. J. Barrett D. A. (2008). Characterisation of the cannabinoid receptor system in synovial tissue and fluid in patients with osteoarthritis and rheumatoid arthritis. Arthritis Res. Ther. 10, R43. 10.1186/ar2401 18416822 PMC2453762

[B40] Russo E. B. (2011). Taming THC: potential cannabis synergy and phytocannabinoid-terpenoid entourage effects. Br. J. Pharmacol. 163, 1344–1364. 10.1111/j.1476-5381.2011.01238.x 21749363 PMC3165946

[B41] Russo E. B. Burnett A. Hall B. Parker K. K. (2005). Agonistic properties of cannabidiol at 5-HT1a receptors. Neurochem. Res. 30, 1037–1043. 10.1007/s11064-005-6978-1 16258853

[B42] Schneider T. Zurbriggen L. Dieterle M. Mauermann E. Frei P. Mercer-Chalmers-Bender K. (2022). Pain response to cannabidiol in induced acute nociceptive pain, allodynia, and hyperalgesia by using a model mimicking acute pain in healthy adults in a randomized trial (CANAB I). Pain 163, e62–e71. 10.1097/j.pain.0000000000002310 34086631

[B43] Sepulveda D. E. Vrana K. E. Kellogg J. J. Bisanz J. E. Desai D. Graziane N. M. (2024). Special section: cannabinoid signaling in human health and disease — minireview the potential of cannabichromene (CBC) as a therapeutic agent. J. Pharmacol. Exp. Ther. 391, 206–213. 10.1124/jpet.124.002166 38777605 PMC11493452

[B44] Shustorovich A. Corroon J. Wallace M. S. Sexton M. (2024). Biphasic effects of cannabis and cannabinoid therapy on pain severity, anxiety, and sleep disturbance: a scoping review. Pain Med. (United States) 25, 387–399. 10.1093/pm/pnae004 38268491

[B45] Vaughn D. M. Paulionis L. J. Kulpa J. E. (2021). Randomized, placebo-controlled, 28-day safety and pharmacokinetics evaluation of repeated oral cannabidiol administration in healthy dogs. Am. J. Vet. Res. 82, 405–416. 10.2460/ajvr.82.5.405 33904801

[B54] Vela J. Dreyer L. Petersen K. K. Arendt-Nielsen L. Duch K. S. Kristensen S. (2022). Cannabidiol treatment in hand osteoarthritis and psoriatic arthritis: a randomized, double-blind, placebo-controlled trial. Pain 163, 1206–1214. 10.1097/j.pain.0000000000002466 34510141

[B46] Verrico C. D. Wesson S. Konduri V. Hofferek C. J. Vazquez-Perez J. Blair E. (2020). A randomized, double-blind, placebo-controlled study of daily cannabidiol for the treatment of canine osteoarthritis pain. Pain 161, 2191–2202. 10.1097/j.pain.0000000000001896 32345916 PMC7584779

[B47] Vos T. Allen C. Arora M. Barber R. M. Brown A. Carter A. (2016). Global, regional, and national incidence, prevalence, and years lived with disability for 310 diseases and injuries, 1990–2015: a systematic analysis for the global burden of disease study 2015. Lancet 388, 1545–1602. 10.1016/S0140-6736(16)31678-6 27733282 PMC5055577

[B48] Vučkovic S. Srebro D. Vujovic K. S. Vučetic Č. Prostran M. (2018). Cannabinoids and pain: new insights from old molecules. Front. Pharmacol. 9, 1–19. 10.3389/fphar.2018.01259 30542280 PMC6277878

[B49] Wallace M. S. Marcotte T. D. Umlauf A. Gouaux B. Atkinson J. H. (2015). Efficacy of inhaled cannabis on painful diabetic neuropathy. J. Pain 16, 616–627. 10.1016/j.jpain.2015.03.008 25843054 PMC5152762

[B50] Wen Y. Wang Z. Zhang R. Zhu Y. Lin G. Li R. (2023). Biomedicine & Pharmacotherapy The antinociceptive activity and mechanism of action of cannabigerol. Biomed. Pharmacother. 158, 114163. 10.1016/j.biopha.2022.114163 36916438

[B51] Wiley J. L. Burston J. J. Leggett D. C. Alekseeva O. O. Razdan R. K. Mahadevan A. (2005). CB1 cannabinoid receptor-mediated modulation of food intake in mice. Br. J. Pharmacol. 145, 293–300. 10.1038/sj.bjp.0706157 15778743 PMC1576140

[B52] Wise B. L. Niu J. Zhang Y. Wang N. Jordan J. M. Choy E. (2010). Psychological factors and their relation to osteoarthritis pain. Osteoarthr. Cartil. 18, 883–887. 10.1016/j.joca.2009.11.016 20346403 PMC2912218

[B53] Yanes J. A. McKinnell Z. E. Reid M. A. Busler J. N. Michel J. S. Pangelinan M. M. (2019). Effects of cannabinoid administration for pain: a meta-analysis and meta-regression. Exp. Clin. Psychopharmacol. 27, 370–382. 10.1037/pha0000281 31120281 PMC6663642

